# “环境暴露组学与生命健康专辑”引言

**DOI:** 10.3724/SP.J.1123.2024.01009

**Published:** 2024-02-08

**Authors:** Xinyu LIU, Surong MEI, Guowang XU

随着工农业的快速发展和生活水平的日益提高,环境污染问题及对人类生命健康的影响越来越受到关注。为了更好地回答“环境和人体中存在哪些污染物,这些‘暴露’因素如何影响健康”等一系列科学问题,暴露组学(exposomics)应运而生。暴露组学关注个体一生所有暴露的测量及这些暴露如何与疾病建立联系,代谢组学(metabolomics)可从代谢水平阐明暴露对健康的影响,发现疾病风险因子和疾病标志物,二者结合促进了以组学为手段的暴露-疾病关系的研究,能很好地探讨污染暴露、生命健康和疾病发生的内在本质。

色谱-质谱联用技术是复杂体系中环境污染物内外暴露测量及代谢组学等多种生命组学分析的主流技术。近年来科研人员利用色谱-质谱平台,建立了一系列暴露组学、代谢组学新技术、新方法,为环境暴露与生命健康研究提供了重要的技术支撑。为了总结当前环境暴露组学与代谢组学的相关研究进展,展望未来研究方向,受《色谱》期刊委托,我们组织了“环境暴露组学与生命健康”专辑,邀请了国内相关高等院校和科研院所等相关领域专家学者为本刊撰稿。

本专辑由1篇视角、4 篇专论与综述、7 篇研究论文和1 篇研究快报组成,涉及环境暴露组监测及健康危害评价的分析研究进展、多种新污染物(包括全氟及多氟烷基化合物、酚类化合物、短链氯化石蜡、抗生素等)和典型污染物(有机氯农药、多氯联苯、多环芳烃等)的靶向/复合暴露监测及健康关联研究。本专辑中的研究工作涉及各种人群,包括孕妇、捐精人群、青少年、普通成人等,监测的人群生物样本基质多样,包括血清、母乳、尿样等。我们衷心希望通过本专辑的出版,推动色谱-质谱联用技术在环境暴露与生命健康交叉领域的广泛应用,为促进“健康中国2030”中营造绿色安全的健康环境的预期目标做出贡献。

衷心感谢各位作者及审稿专家的鼎力支持和奉献!

本专辑客座主编

中国科学院大连化学物理研究所 刘心昱 副研究员

华中科技大学公共卫生学院环境医学研究所 梅素容 教授

中国科学院大连化学物理研究所 许国旺 研究员

2024年1月10日

